# Energy drink use, problem drinking and drinking motives in a diverse sample of Alaskan college students

**DOI:** 10.3402/ijch.v72i0.21204

**Published:** 2013-08-05

**Authors:** Monica C. Skewes, Christopher R. DeCou, Vivian M. Gonzalez

**Affiliations:** 1 Department of Psychology, University of Alaska Fairbanks, Fairbanks, AK, USA; 2 Center for Alaska Native Health Research, University of Alaska Fairbanks, Fairbanks, AK, USA; 3 Department of Psychology, Idaho State University, Pocatello, ID, USA; 4 Department of Psychology, University of Alaska Anchorage, Anchorage, AK, USA

**Keywords:** caffeine, alcohol problems, drinking to cope

## Abstract

**Background:**

Recent research has identified the use of caffeinated energy drinks as a common, potentially risky behavior among college students that is linked to alcohol misuse and consequences. Research also suggests that energy drink consumption is related to other risky behaviors such as tobacco use, marijuana use and risky sexual activity.

**Objective:**

This research sought to examine the associations between frequency of energy drink consumption and problematic alcohol use, alcohol-related consequences, symptoms of alcohol dependence and drinking motives in an ethnically diverse sample of college students in Alaska. We also sought to examine whether ethnic group moderated these associations in the present sample of White, Alaska Native/American Indian and other ethnic minority college students.

**Design:**

A paper-and-pencil self-report questionnaire was completed by a sample of 298 college students. Analysis of covariance (ANCOVA) was used to examine the effects of energy drink use, ethnic group and energy drink by ethnic group interactions on alcohol outcomes after controlling for variance attributed to gender, age and frequency of binge drinking.

**Results:**

Greater energy drink consumption was significantly associated with greater hazardous drinking, alcohol consequences, alcohol dependence symptoms, drinking for enhancement motives and drinking to cope. There were no main effects of ethnic group, and there were no significant energy drink by ethnic group interactions.

**Conclusion:**

These findings replicate those of other studies examining the associations between energy drink use and alcohol problems, but contrary to previous research we did not find ethnic minority status to be protective. It is possible that energy drink consumption may serve as a marker for other health risk behaviors among students of various ethnic groups.

The use of caffeinated energy drinks worldwide has grown substantially in recent years and has raised important questions concerning the potential health impacts of this growing trend (1). Recent investigations have identified the use of energy drinks as a common and potentially risky behavior among college students (2–5). Although the problematic use of energy drinks is not limited exclusively to college students (6), the college setting and lifestyle in combination with targeted marketing of energy drinks towards adolescents and emerging adults may contribute to high rates of energy drink use among students. Associations have been noted between the consumption of energy drinks and health risk behaviors among college students (1). Recent scholarship has identified numerous examples of such associations, including increased alcohol consumption, alcohol-related consequences, sexual risk-taking, tobacco use and the use of other substances (2,4,7–9).

Several studies have noted the association between energy drink and alcohol misuse among college students (2,4,7). This association has been particularly salient in research that specifically measured consumption of alcohol mixed with energy drinks (AMED; 2,9). A recent study found that college students consuming AMED reported increased alcohol consumption compared to other drinking episodes during which they had not mixed alcohol with energy drinks, after controlling for respondents’ self-reported risk-taking propensity (2). A recent experiment found increased self-reported desire to consume alcohol among participants randomly assigned to drink AMED in comparison to alcohol-only, energy drink-only and placebo conditions (10). These findings are congruent with previous research highlighting the association between energy drink use and alcohol consequences.

Specifically, O'Brien et al. (7) conducted a web-based survey of college students (N=4,271), and observed substantially greater rates of problem drinking behaviors (e.g. drunkenness, heavy episodic drinking) in AMED users compared to students who did not combine alcohol with energy drinks (7). These findings, among others (e.g. 9,11,12), demonstrate the problematic consequences of combining alcohol and energy drinks, and may indicate a moderating effect of energy drink use on the relationship between alcohol consumption and alcohol-related problems among college students. However, research focused specifically on AMED does not address the broader associations that may exist between overall energy drink consumption (i.e. not mixed with alcohol as a cocktail) and other health risk behaviors and mental health variables.

Other recent research evaluated data from a longitudinal study of university undergraduates and found that the association between energy drinks and alcohol consumption appears to be related to specific patterns (i.e. low-dose or high-dose) of energy drink consumption (13). The authors found that energy drink consumption was predictive of alcohol consumption for those students engaging in daily or weekly consumption of energy drinks. Conversely, no such association was observed among students who reported “low-dose” consumption of energy drinks, even after controlling for family history of alcohol problems and typical alcohol consumption (13).

As distinguished from investigations that have explored AMED specifically, Miller identified an association between overall frequency of energy drink consumption and problem drinking behaviors among college students (4). Furthermore, Miller observed associations between the frequency of energy drink use and the use of tobacco, marijuana, non-medical use of prescription medications and sexual risk-taking (4). However, the use of energy drinks did not predict risk-taking as measured by participation in extreme sports. In this way it seems that the frequency of energy drink consumption may not predict all types of risk-taking, but rather explains certain health risk behaviors, like binge drinking, that are normative within the context of university communities. Additionally, Miller found that race moderated the relationship between energy drinks and substance use; that is, the associations between energy drinks and substance use observed among White college students were not observed among African American students (14). Similarly, O'Brien et al. observed variation in university students’ use of AMED, with White students evidencing greater likelihood of AMED use (7). These findings raise the question of whether ethnicity may serve as a protective factor for negative outcomes related to energy drink use among other ethnic minorities.

The present study analysed data from a survey of 298 college students at a minority-serving 4-year public university in Alaska. The survey included various measures of health-related behaviors including energy drink consumption, alcohol involvement, other substance use and mental health variables. This paper specifically addresses the relationship between self-reported weekly energy drink consumption and measures of hazardous drinking, alcohol consequences, symptoms of alcohol dependence and drinking motives. Although there is a growing literature concerning the health effects of energy drinks, this paper seeks to contribute to the existing literature in the following ways: (a) replicate findings of previous research concerning the relationship between energy drinks and problematic alcohol use in an ethnically diverse sample of Alaskan university students; (b) examine the associations between energy drink consumption and drinking motives; and (c) examine the effect of ethnic group on these associations in our sample of White, Alaska Native/American Indian and other ethnic minority college students.

## Method

### Participants

Participants included 298 undergraduate college students (63.4% female) enrolled at a public university in the circumpolar north. Ages ranged from 18 to 52 (M=23.03,SD=6.53). The majority of students were emerging adults (80.1% were between 18 and 25 years of age). Freshmen were most represented in our sample (n=125, 41.9%), with the remainder of the sample evenly distributed between sophomores, juniors and seniors. Regarding ethnicity, 68.4% (n=201) students self-identified as White, 17.7% (n=52) identified as Alaska Native or American Indian, and 13.9% (n=45) were other ethnic minorities. Participants in the “other” category included African American, Latino/a and Asian American students. The characteristics of the current sample were reasonably aligned with population characteristics, as 59% of the university's student body is female, the median age is 25, and approximately 18% of the student body is Alaska Native/American Indian.

### Measures

#### Energy drink use

Energy drink consumption was measured using a single item instructing participants to indicate the number of energy drinks they consume per week, on average. Five categories ranging from 0 (*less than one*) to 5 (*15 or more*) were provided for respondents. Examples of energy drinks were provided so as not to be confused with coffee or other caffeinated beverages. Energy drinks consumed per average week were used as an independent variable in the analyses.

#### Hazardous drinking

The Alcohol Use Disorders Identification Test (AUDIT; 15) is a 10-item self-report instrument developed by the World Health Organization as a screening tool for hazardous drinking. Items are scored from 0 to 4 and responses are summed to yield a total score, with higher values indicating a greater likelihood of having an alcohol use disorder. AUDIT scores ≥8 indicate the presence of “hazardous drinking” (16).

One item asked respondents to indicate the frequency with which they consume 6 or more standard drinks on 1 occasion, rated from 0 (never) to 4 (4 or more times per week). As binge drinking is currently defined as 4 or more standard drinks in 1 sitting for a woman and 5 or more standard drinks for a man (17), this item provides a conservative estimate of binge drinking for both women and men. Frequency of binge drinking was included as a covariate in the analyses.

#### Alcohol problems

The Young Adult Alcohol Consequences Questionnaire (YAACQ; 18) is a 48-item self-report inventory of problems commonly associated with alcohol use among college students. Items are rated as: present (1) or absent (0) within the past 12 months. Responses are summed to yield a total score, with higher scores indicating greater alcohol problems.

#### Alcohol dependence symptoms

The Short Alcohol Dependence Data questionnaire (SADD; 19) is a 15-item measure of current symptoms of alcohol dependence such as excessive thinking about drinking or perceived inability to control one's drinking. Items are scored from 0 (*never*) to 3 (*nearly always*) and are summed to yield a total score, with higher scores indicating greater severity of dependence symptoms.

#### Drinking motives

The Drinking Motives Questionnaire-Revised (DMQ-R; 20) consists of four 5-item subscales assessing the motives for drinking, including drinking for social reasons (e.g. to bond with others), drinking to cope with negative affect (e.g. to forget one's problems), drinking for enhancement reasons (e.g. to increase enjoyment or have fun) and drinking for conformity reasons (e.g. drinking to fit in). Questions ask respondents to indicate how frequently they drink in response to these motives on a scale of 1 (*almost never*) to 4 (*almost always*). The possible range for each subscale (Social, Coping, Enhancement, Conformity) is 5–20, with higher scores indicating greater self-reported drinking in response to that motive.

#### Demographics

Participants were instructed to indicate their age, gender and ethnicity. Participants who self-reported American Indian or Alaska Native heritage were classified as “Native.” The remaining participants were classified by self-report as “White” or “Other ethnic minority.” The “Other” group included an approximately even distribution of African American, Latino/a and Asian American participants.

### Procedure

Participants were recruited via in-class announcements and received extra course credit for their time. They gave informed consent and completed packets of paper-and-pencil measures in small groups. Each participant was given a manila envelope with the survey and instructed to place the completed questionnaire into the envelope and then to place the envelope into a large box filled with other similar envelopes. This procedure was intended to enhance participants’ confidence that their responses would not be linked back to them or identifiable in any way, thereby improving validity of self-reports. The university's Institutional Review Board approved all methods and materials.

### Analyses

Descriptive statistics and bivariate correlations were calculated for all study variables. Then, a series of analyses of covariance (ANCOVAs) were conducted to examine the effects of ethnic group and energy drink consumption on alcohol outcomes while controlling for variance attributed to age, gender and frequency of binge drinking. The independent variables in all analyses were ethnic group and energy drink consumption. The covariates were age, gender and frequency of binge drinking. The dependent variables were AUDIT scores, YAACQ scores, SADD scores and all DMQ subscale scores. Main effects of ethnic group and energy drink consumption were examined and are reported here. Interactions between ethnicity and energy drink use were examined, but because there were no significant interactions, only main effects will be discussed.

## Results

### Descriptives and correlations

In the present sample, 39.2% of participants (n=124) reported consuming energy drinks at least once per week, with the number of energy drinks per week ranging from 0 to 15+. The majority of the sample reported consuming alcohol in the previous 30 days (66.3%; n=191), and the mean number of standard drinks consumed in the prior month was 12.61 (SD=19.11, range=0–88). Total AUDIT scores ranged from 0 to 25 (M=5.23, SD=4.98) with 31.9% of women and 18.8% of men meeting the criteria for hazardous drinking. Frequency of consuming 6+ standard drinks on 1 occasion ranged from 0 to 16 days per month (M=1.16, SD=2.91).

Total YAACQ ranged from 0 to 48 (M=8.83, SD=10.57) and SADD scores ranged from 0 to 22 (M=3.13, SD=4.40). The full range (0–20) for all DMQ subscales was present, with the highest scores reported for social motives (M=11.18, SD=4.65), followed by enhancement motives (M=10.09, SD=4.45), coping motives (M=8.87, SD=4.01) and conformity motives (M=6.65, SD=2.71). Energy drink consumption was significantly negatively associated with age (r=−16, p<.01), but was not associated with gender. There were no significant differences in energy drink use by ethnic group. Significant associations were found between energy drink consumption and all dependent variables with the exception of the DMQ-conformity subscale scores (see Table I for bivariate correlations between energy drink use and all dependent variables).

**Table I:** Intercorrelations of dependent variables and energy drink consumption (N=298)

Variables	1	2	3	4	5	6	7	8
1. Hazardous drinking	—							
2. Alcohol consequences	.74***	—						
3. Dependence symptoms	.79***	.75***	—					
4. Motives – social	.62***	.52***	.54***	—				
5. Motives – coping	.64***	.63***	.65***	.76***	—			
6. Motives – enhancement	.66***	.55***	.63***	.79***	.74***	—		
7. Motives – conformity	.24***	.31***	.25***	.52***	.47***	.35***	—	
8. Energy drinks per week	.25***	.15*	.17**	.18**	.24***	.28***	.02	—

* Note: p<.05

** p<.01

*** p<.001

### Analyses of covariance

See Table II for the marginal means of the dependent variables examined in the ANCOVAs by ethnic group. Controlling for age, gender and frequency of binge drinking, energy drink use was a significant predictor of total AUDIT scores (F(4, 257)=6.00, p<.001, partial η^2^=.09) but there were no significant differences in AUDIT scores between ethnic groups, nor was there a significant energy drink by ethnic group interaction. Similarly, in the ANCOVA examining YAACQ scores, energy drink use was a significant predictor (F(4, 257)=2.66, p<.05, partial η^2^=.04) but ethnic group was not. There also was no significant interaction. This pattern of findings was observed for SADD scores also, with energy drink use emerging as a significant predictor (F(4, 249)=3.75, p<.01, partial η^2^=.06), while ethnic group and the energy drink by ethnic group interaction were not significant.

**Table II:** Adjusted (marginal) means and standard deviations for 3 ethnic groups and 7 dependent variables (N =298)

Variables	White	Alaska Native	Other ethnic minority
1. Hazardous drinking	6.42 (.44)	5.39 (.77)	8.95 (1.10)
2. Alcohol consequences	10.68 (1.18)	11.44 (2.12)	12.41 (2.94)
3. Dependence symptoms	3.77 (.47)	3.81 (.83)	6.58 (1.17)
4. Motives – social	12.43 (.56)	11.37 (1.00)	12.89 (1.39)
5. Motives – coping	10.03 (.47)	9.71 (.85)	11.79 (1.18)
6. Motives – enhancement	11.49 (.50)	10.93 (.91)	13.44 (1.26)
7. Motives – conformity	6.23 (.34)	8.35 (.79)	6.48 (.87)

ANCOVAs also were conducted to examine each drinking motive as measured by the DMQ-R, controlling for age, gender and binge drinking. None of the independent variables significantly predicted DMQ-Social or DMQ-Conformity scores, although the binge drinking covariate was significant for both. Energy drink use was a significant predictor of DMQ-Coping scores (F(4, 249)=5.04, p=.001, partial η^2^=.08), but neither ethnic group nor the energy drink by ethnic group interaction were significant. Energy drink use also significantly predicted DMQ-Enhancement scores (F(4, 248)=4.60, p=.001, partial η^2^=.07)

## Discussion

This research sought to replicate the previously reported associations between energy drink use, problem drinking and alcohol consequences in an ethnically diverse sample of Alaskan college students. Further, we explored energy drink use in relation to alcohol dependence symptoms and drinking motives. Because of Miller's finding that race moderated the association between energy drink consumption and alcohol outcomes in a sample of White and African American college students (4), we examined energy drink by ethnic group interactions in the present sample. Contrary to Miller's research, we found no moderating effect of ethnicity, nor did we find a main effect of ethnic group for any of the dependent variables examined. In the present sample, the associations between energy drink use and alcohol outcomes were equivalent for White, Alaska Native/American Indian and other ethnic minority Alaskan students.

There were several significant and moderate correlations between variables. Students who reported greater energy drink consumption also reported greater frequency of binge drinking, greater drinking to cope, greater alcohol-related problems, greater alcohol dependence symptoms, and were at greater risk of having an alcohol use disorder. Controlling for age, gender and frequency of binge drinking, energy drink use significantly predicted scores on measures of problem drinking, alcohol consequences, dependence symptoms, enhancement motives and coping motives. Of particular interest was the association between energy drink use and coping drinking motives. Drinking to cope is a particularly problematic motive that has been shown to predict greater alcohol use and greater severity of alcohol problems and consequences than drinking for other motives (21,22). Beyond its influence on alcohol use itself, drinking to cope may affect decision making regarding when and where to drink, thereby increasing alcohol consequences above and beyond its direct effect on alcohol consumption (23).

These findings, along with those from previous research, raise the question: why does energy drink use predict alcohol problems after controlling for alcohol consumption? We do not posit that the link is causal. However, we do hypothesize that intrapersonal factors such as personality variables (e.g. sensation seeking) may account for substance use behavior in general, including energy drink use. Indeed, other research has found greater sensation seeking among energy drink users than non-energy drink users (8,24). Further research is needed to understand the nature of these relationships – particularly if energy drink use may serve as a marker for other problem behaviors and symptoms.

In addition to our hypothesis regarding individual difference variables explaining the association between energy drinks and alcohol, we also think it is possible that some students may be self-medicating for untreated symptoms of Attention Deficit Hyperactivity Disorder (ADHD). Stimulants are used to treat ADHD symptoms, and caffeine may improve concentration and focus among people with ADHD. Although some research has examined motives for consuming AMED (e.g. 11) future research should examine motives for energy drink use independent of AMED as well as energy drink outcome expectancies among college students. A promising measure of caffeine expectancies has recently been developed (i.e. Caffeine Expectancy Questionnaire; 25) and would yield important information for understanding energy drink motives in future research.

### Limitations

Limitations of this research include the overrepresentation of women and the underrepresentation of Alaska Native/American Indian and other ethnic minority students in the current sample. It is entirely possible that ethnicity may serve as a protective factor for minority students but that the small percentages of non-White students in this sample did not allow us to detect significant main effects or interactions. It is possible that African American culture is protective (4), but because African American students were grouped with other ethnic minorities the protective effects were obscured. Unfortunately, we did not have the statistical power to examine African American students separately from other ethnic minorities. Future research should use purposive sampling to replicate Miller's finding that energy drink use was not associated with problematic drinking and alcohol consequences among African American students (4). Other limitations include the cross-sectional design of this research, which does not allow for examination of causal pathways. Moreover, we neglected to assess whether energy drinks were being consumed with alcohol in AMED or without alcohol, which may have facilitated deeper understanding of the link between energy drink use and alcohol consumption. Finally, we did not assess sensation seeking, an important personality variable that has been shown to differ between energy drink users and non-users (8,24).

Nevertheless, this research contributes to the extant literature on this topic by examining the effect of overall energy drink use on various criterion variables related to problematic alcohol use, including the important alcohol motive of drinking to cope. This research also is unique in its focus on a college student sample in the circumpolar north with high rates of alcohol use disorders. As research examining the effects of energy drink use on risky behaviors and health outcomes accumulates, we will be able to better understand how to use knowledge about college students’ energy drink consumption to identify students at risk for serious problems.
